# Identification of novel common variants associated with chronic pain using conditional false discovery rate analysis with major depressive disorder and assessment of pleiotropic effects of *LRFN5*

**DOI:** 10.1038/s41398-019-0613-4

**Published:** 2019-11-20

**Authors:** Keira J. A. Johnston, Mark J. Adams, Barbara I. Nicholl, Joey Ward, Rona J. Strawbridge, Andrew M. McIntosh, Daniel J. Smith, Mark E. S. Bailey

**Affiliations:** 1 Institute of Health and Wellbeing, University of Glasgow, Scotland, UK; 2 Deanery of Molecular, Genetic and Population Health Sciences, College of Medicine and Veterinary Medicine, University of Edinburgh, Scotland, UK; 3 School of Life Sciences, College of Medical, Veterinary & Life Sciences, University of Glasgow, Scotland, UK; 4 Division of Psychiatry, University of Edinburgh, Scotland, UK; 5 Department of Medicine Solna, Karolinska Institute, Stockholm, Sweden

**Keywords:** Personalized medicine, Depression, Comparative genomics

## Abstract

Chronic pain is a complex trait that is moderately heritable and genetically, as well as phenotypically, correlated with major depressive disorder (MDD). Use of the conditional false discovery rate (cFDR) approach, which leverages pleiotropy identified from existing GWAS outputs, has been successful in discovering novel associated variants in related phenotypes. Here, genome-wide association study outputs for both von Korff chronic pain grade and for MDD were used to identify variants meeting a cFDR threshold for each outcome phenotype separately, as well as a conjunctional cFDR (ccFDR) threshold for both phenotypes together. Using a moderately conservative threshold, we identified a total of 11 novel single nucleotide polymorphisms (SNPs), six of which were associated with chronic pain grade and nine of which were associated with MDD. Four SNPs on chromosome 14 were associated with both chronic pain grade and MDD. SNPs associated only with chronic pain grade were located within *SLC16A7* on chromosome 12. SNPs associated only with MDD were located either in a gene-dense region on chromosome 1 harbouring *LINC01360*, *LRRIQ3, FPGT* and *FPGT-TNNI3K*, or within/close to *LRFN5* on chromosome 14. The SNPs associated with both outcomes were also located within *LRFN5*. Several of the SNPs on chromosomes 1 and 14 were identified as being associated with expression levels of nearby genes in the brain and central nervous system. Overall, using the cFDR approach, we identified several novel genetic loci associated with chronic pain and we describe likely pleiotropic effects of a recently identified MDD locus on chronic pain.

## Introduction

Chronic pain is defined as pain lasting longer than 12 weeks. It affects around 30% of the global adult population^1^, imposes significant socioeconomic burden, and contributes to excess mortality^2,3^. Chronic pain is often associated with both specific and non-specific medical conditions (such as cancers and HIV/AIDS, fibromyalgia and musculoskeletal conditions), and with a range of injuries^4,5^. It is also commonly co-morbid with mood disorders, such as major depressive disorder (MDD)^6–8^. MDD is a common and serious mood disorder involving psychological symptoms such as persistent low mood and anhedonia, and physical symptoms such as changes in appetite and sleep disturbance (American Psychiatric Association 2013). In common with chronic pain, MDD also imposes a significant socioeconomic burden worldwide and is now the leading cause of disability globally^2^. The causal direction of factors underlying the association between chronic pain and mood disorders is unclear.

### Genetics of chronic pain and related disorders

The genetics of pain have hitherto mostly been investigated using candidate gene and animal-model approaches^9,10^. Several aspects of chronic pain, such as chronic pain grade (a graded classification of chronic pain assessed using a questionnaire first constructed by von Korff and colleagues^11^ and further validated by Smith et al.^12^), pain at specific sites (e.g. back pain), and specific chronic-pain-related conditions such as migraine and temporomandibular joint (TMJ) disorder, have been shown to be complex traits with moderate heritability and associated genetic variants have been discovered^3,7,9,10,13–15^. In order to be suitably well-powered genome-wide association studies of complex traits (such as chronic pain) must be of sufficient sample size (>~2000 individuals), amongst other restrictions (see Hong et al.^16^). However, in part due to the heterogeneity of pain assessment and pain experience^17^, there are very few large-scale genetic studies of chronic pain as a phenotype in its own right irrespective of site, inciting injury or condition^9,10,14,18^ and no genome-wide significant genetic variants have yet been identified for chronic pain grade specifically.

Chronic pain and chronic pain disorders are often comorbid with psychiatric and neurodevelopmental disorders, including MDD^19^. MDD has also been shown to be genetically correlated with chronic pain grade specifically (*r*_g _= 0.53^7^), and shared genetic factors have been reported between some specific chronic pain conditions and MDD^20–22^. Chronic pain and depression can also be grouped together into ‘Chronic Pain Syndromes’, a poorly-defined range of syndromes involving chronic pain and exhibiting genetic overlap^23^.

The immune and nervous systems play a central role in chronic pain development and maintenance^24,25^. Similarly, obesity and chronic pain are often comorbid, with extrinsic factors such as sleep disturbance also impacting on chronic pain^26,27^. Altered sleep quality and reduced circadian rhythmicity are also common in those with chronic pain^28^. Chronic pain can also be commonly reported in those with neurological diseases^29^.

### Chronic pain phenotyping

The relationship between injury and other peripheral insult, consequent acute pain and the subsequent development of chronic pain has not been fully explained. Not everyone who undergoes major surgery or is badly injured will develop chronic pain, for example^30^, and the degree of joint damage in osteoarthritis often does not match the severity of chronic pain experienced^31^. Conversely, complex regional pain syndrome (CRPS) can in rare cases be incited by minor peripheral insult such as insertion of a needle^32–34^. Structural and functional changes in the brain and spinal cord are associated with the development and maintenance of chronic pain, such as aberrant feedback between the spinal cord and brain^30,35,36^, and affective brain regions are involved in chronic pain perception^37–41^. It is also unlikely that there are legitimate cut-off points or thresholds for localised and widespread chronic pain, with chronic pain instead existing on a continuous spectrum^42^. Thus, exploration of the molecular mechanisms underlying chronic pain is likely to benefit from a focus on pain phenotypes relating to chronic pain as a neuropathological trait in its own right.

### Conditional false discovery rate (cFDR) analyses

An alternative strategy for identifying genetic variation contributing to complex traits is to make use of existing GWAS summary statistic outputs within more powerful discovery approaches, such as the cFDR approach^43^. The cFDR approach makes better use of the genetic overlap between traits or conditions to discover loci based on their pleiotropic effects in each phenotype separately, as the strength of evidence for involvement of a locus in a primary trait may be enhanced by consideration of the strength of evidence for the same locus in an associated secondary trait. In the case of chronic pain, the association with mood disorders and mood-related traits is of substantial interest, as improved understanding of the biological underpinnings of this overlap may stimulate the development of novel treatment strategies.

cFDR analyses have been used to find novel variants associated with schizophrenia, type 2 diabetes, Alzheimer disease, bipolar disorder and systolic blood pressure^43–46^. This therefore represents a promising and potentially more cost-effective method for identifying new single nucleotide polymorphisms (SNPs) associated with complex traits by maximising the utility of existing GWAS outputs.

In the study reported here we aimed to find chronic pain-associated SNPs using cFDR analysis, with MDD as the secondary trait. In addition, we sought to discover loci with pleiotropic effects on these two phenotypes. We have chosen to focus on an aspect of chronic pain related to pain severity—chronic pain grade (CPG).

The power to detect CPG-associated SNPs was increased by leveraging the additional information coming from the correlation between predisposing loci for CPG and for MDD. Overall, we found six CPG-associated and nine MDD-associated SNPs. In addition, pleiotropic effects of four *LRFN5* SNPs were observed. These findings contribute to our understanding of the biological underpinnings of chronic pain and a component of its shared biology with MDD.

## Methods

### Phenotype definition and source data

For chronic pain in the cFDR analysis, summary statistics from a GWAS carried out collaboratively with Pfizer-23andMe Inc^7^ were used. Briefly, chronic pain severity was assessed via CPG;^11^, on a scale from 0 (no chronic pain reported) to 4 (high intensity, high disability, severely limiting chronic pain). Characteristic pain intensity, days in pain, time since onset, disability score and disability days were quantified and used to calculate a CPG category per individual^11^, through a questionnaire consisting of seven question items.

The GWAS of CPG was then carried out by 23andMe in collaboration with Pfizer, using data from 23 301 unrelated individuals of white European descent (10 543 individuals with CPG levels 1–4 combined, 12 758 individuals with CPG level 0; Supplementary Table 1). For MDD, summary statistics from a recent case-control GWAS meta-analysis^47^ were provided by the Psychiatric Genomics Consortium (PGC) after removal of data from 23andMe and UK Biobank participants, yielding a dataset originating from an analysis using 43,028 cases and 87,522 controls. Thus, none of the participants contributing to the CPG summary statistics overlapped with those contributing to the MDD summary statistics. Phenotype definitions, study population demographics and meta-analysis procedures for the MDD GWAS have been described previously^47^.

### Data preparation and LD pruning

A dataset of SNPs for which a *p*-value for association, chromosome position data and rsID were available in both MDD and CPG datasets was compiled. This was then LD pruned as follows. Firstly, PLINK-format genotype data, for each SNP in the newly compiled CPG-MDD summary-statistic dataset, was extracted from the UK Biobank genotype data (approved application 6553). Pruning was carried out using command line PLINK (version 1.9)—indep-pairwise function. From each SNP-pair with *r*^2^ > 0.2 within a 50-SNP window of the lead SNP, one was removed according to PLINK’s greedy pruning algorithm, the window slid along 5 SNPs, and the pruning repeated. This resulted in an LD-pruned dataset of 774,292 SNPs with association data available for both MDD and CPG.

### cFDR analyses

cFDR is an extension of local-area false-discovery rate (FDR), which is in turn a re-thinking of Benjamini & Hochberg’s ‘tail-end’ false discovery rate procedure^48–51^. The tail-end FDR procedure is concerned with controlling the FDR at a pre-defined level and deciding the maximum test statistic value from a list of ordered test statistic values that allows for this^49^. Local FDR reframes the FDR as a Bayesian posterior probability that the SNP in question is not associated with the disease or trait, given its association test statistic^50,51^.

cFDR analysis of GWAS summary statistics extends local FDR, with the result being the posterior probability that the SNP in question is not associated with the primary phenotype, given its association test statistic value for both the primary phenotype and for a secondary, related phenotype. This allows for exploitation of any underlying pleiotropic genetic architecture, believed to be ubiquitous in human complex disease^52,53^.

cFDR and conjunctional cFDR (ccFDR) values for each SNP for CPG given MDD and vice versa were calculated (Formula 1) as previously detailed^48^ using the statistical software R (version 3.3.3).

1 $${\mathrm{cFDR}} = \Pr \left( {H_{0\left( i \right)}\left| {p_i \le P_i,p_j \le P_j} \right.} \right) = \frac{{p_i}}{{\Pr \left( {p_i \le P_i\left| {p_j \le P_j} \right.} \right)}}$$

The cFDR value for a SNP obtained from the above formula is the probability that the SNP is not associated with the primary phenotype, given its strength of association with the secondary phenotype. The ccFDR was obtained via taking the higher of the two cFDR values obtained for each SNP. The significance threshold chosen was cFDR (and ccFDR) ≤ 0.01.

### Genomic context analyses

The genomic context for SNPs with significant cFDR values in either analysis was examined. The R package ‘rsnps’ was used to extract data from records in NCBI dbSNP (https://www.ncbi.nlm.nih.gov/snp). Gene context for each SNP was examined in the UCSC Genome Browser (build GRCh38/hg38)^54^, using a window of 0.5Mbp around each SNP and data from the GENCODE v24 track, validated or reviewed by either Refseq or SwissProt staff. The presence of cis-eQTLs close to the significant SNPs was investigated using the IGV eQTL Browser^55^ web interface.

### Further exploration of pleiotropy in *LRFN5*

Regression models were run to examine associations between genotype at a SNP identified as pleiotropic in the ccFDR analysis and relevant phenotypes in data from the UK Biobank (chronic pain, MDD, age attended assessment centre and sex; approved applications 10302, 6553). Genotype at rs11846556 (the SNP with the lowest ccFDR) in UK Biobank individuals was coded as number of effect-associated alleles (0, 1, 2 copies of the A allele).

UK Biobank participants were asked via a touchscreen questionnaire about “pain types experienced in the last month” (field ID 6159) during baseline investigations. Possible answers were: ‘None of the above’; ‘Prefer not to answer’; pain at seven different body sites (head, face, neck/shoulder, back, stomach/abdomen, hip, knee); or ‘all over the body’. The seven body-site pain options were not mutually exclusive, but if a participant chose ‘pain all over the body’ they could not then select any other pain sites. Where patients reported recent pain at one or more body sites, or all over the body, they were additionally asked (category ID 100048) whether this pain had lasted for 3 months or longer.

A dichotomous chronic pain variable was defined as the presence of chronic pain in at least 1 site (or all over the body) for longer than 3 months. Those who did not report pain of that duration were assigned to a control group.

In addition to this dichotomous ‘presence of chronic pain’ variable, a chronic pain variable capturing information about the number of sites of chronic pain was also created (chronic pain category; treated as an ordinal variable in regression models), again employing the 3 month minimum duration criterion: 0 = no chronic pain, 1 = single site, 2 = 2–3 sites, 3 = 4–7 sites, 4 = all over the body (Nicholl et al 2014). Individuals giving ‘prefer not to answer’ and ‘do not know’ answers were excluded from all analyses.

MDD cases were ascertained according to Davis et al.^56^ from a subset of UK Biobank participants who completed a detailed the mental health questionnaire assessment (*n* = 157 366). Controls consisted of those with no mood disorder; those with bipolar disorder were excluded from analyses. An ordinal MDD phenotype (MDD severity) was also obtained by classifying MDD by number of episodes: 0 = no mood disorder, 1 = single episode MDD, 2 = recurrent MDD.

A UK Biobank dataset where all individuals had complete information on age, sex, rs11846556 genotype and chronic pain variables was assembled (*n* = 469 253, Supplementary Table 2). Logistic regression of chronic pain (case-control) on rs1186556 genotype (adjusted for age and sex) and ordinal regression of chronic pain category on rs1186556 genotype (adjusted for age and sex) were then carried out to assess the relationship between SNP genotype and chronic pain.

A second dataset where all individuals had complete information on age, sex, rs11846556 genotype and MDD variables was assembled (*n* = 122,286, Supplementary Table 3). Logistic regression of MDD (case-control) on rs11846556 genotype (adjusted for age and sex), and ordinal regression of MDD severity on rs11846556 genotype (adjusted for age and sex) were then performed to assess the relationship between SNP genotype and MDD.

A final dataset, where all individuals had complete information on age, sex, genotype, chronic pain and MDD variables was assembled (*n* = 121,246, Supplementary Table 4). Logistic regressions of MDD (case-control) on chronic pain (case-control) and genotype (adjusted for age and sex), and of chronic pain (case-control) on MDD (case-control) and genotype (adjusted for age and sex) were then carried out, to assess whether any relationship between MDD and SNP genotype was attenuated by chronic pain, and whether any relationship between SNP genotype and chronic pain was attenuated by MDD.

*P* values for ordinal regression estimates were obtained via comparing the t value obtained to that of a normal t-distribution. Standard errors were used to calculate 95% CI values for ordinal regression output. All models were adjusted for age and sex, both of which were highly significant in all cases. Some models were additionally adjusted for the phenotype (pain or MDD) not being tested as the outcome variable.

## Results

### cFDR and ccFDR analyses

The cFDR approach was used to identify variants associated with CPG, MDD, or both together in a pleiotropic fashion. Eleven SNPs in total were found at cFDR ≤ 0.01 (Table 1), six of which, located on chromosomes 12 and 14, were associated with CPG and nine of which, located on chromosomes 1 and 14, were associated with MDD. Four of these 11 SNPs, all located within a 131 kbp region on chromosome 14, were found to be pleiotropic (ccFDR ≤ 0.01). A lookup table of the top 50 cFDR results, ordered by cFDR value with MDD as the primary trait, is provided in Supplementary Table 5.

**Table 1:** Loci identified from cFDR analysis.

rsID	Position	Alleles	Beta (CPG)	*P* (CPG)	OR (MDD)	*P* (MDD)	cFDR (CPG)	cFDR (MDD)	ccFDR
rs4904790	14:42242623	C/T	−0.03	1.44E-03	1.042	1.37E-05	0.02	3.58E-03	0.02
rs1584317	14:42213816	C/G	0.028	4.48E-03	0.96	4.59E-06	0.029	3.57E-03	0.03
**rs11846556**	14:42183025	A/G	−0.037	1.11E-04	1.05	2.98E-07	5.57E-04	3.76E-05	5.57E-04
**rs10131184**	14:42166111	A/G	0.035	2.82E-04	0.95	2.53E-08	8.46E-04	7.10E-06	8.46E-04
**rs8015100**	14:42095232	A/T	−0.033	6.67E-04	1.06	1.50E-09	6.67E-04	9.27E-07	6.67E-04
**rs11157241**	14:42051771	C/T	0.035	2.91E-04	0.94	4.44E-09	5.83E-04	1.28E-06	5.83E-04
rs10138559	14:41975989	C/T	−0.02	0.03	1.042	1.04E-06	0.1	5.25E-03	0.1
rs10872954	14:41948768	A/G	−0.026	6.45E-03	1.04	7.68E-06	0.053	7.02E-03	0.053
rs149981001	12:60264802	C/T	0.2	6.24E-08	1.087	0.0181	1.06E-03	0.018	0.018
rs147573737	12:60231575	C/T	−0.2	2.09E-07	0.917	0.023	2.21E-03	0.023	0.023
rs35641559	1:73760104	C/T	0.02	0.03	0.961	2.08E-06	0.1	8.83E-03	0.11

Alleles are given as effect allele/other; effect allele is defined as the allele for which association with the trait was tested in the original (CPG or MDD) GWAS. rsIDs for SNPs associated with both MDD and CPG (pleiotropic SNPs) (ccFDR < 0.01) are shown in bold

*Position* position given as chromosome:base pair location, *cFDR (CPG)* cFDR for CPG conditioning on MDD, *Beta (CPG)/p (CPG)* effect size and *p*-value from the CPG GWAS, *cFDR (MDD)* cFDR for MDD conditioning on CPG, *OR (MDD)/p (MDD)* effect size (odds ratio for the effect allele) and *p*-value from the MDD GWAS

### Genomic context

The SNPs associated with CPG only were located close to *SLC16A7* on chromosome 12 (Supplementary Table 6a, 6b), while the SNPs associated with MDD only were located within a gene-dense region on chromosome 1 containing *LINC01360, LRRIQ3, FPGT, FPGT-TNNI3K*, amongst other genes (Supplementary Table 6b) or close to (upstream of) or within *LRFN5* on chromosome 14 (Supplementary Table 6a, 6b). The SNPs found to be pleiotropic were also located within *LRFN5* on chromosome 14 (Supplementary Table 6a, 6b). The pleiotropic *LRFN5* SNPs were all located just upstream of the 5′-most promoter, or within a large intron close to the 5′-end of the gene. The SNPs associated with MDD only were also located within this intron, or were located a little further upstream of the gene. The CPG-only SNPs were all located downstream of *SLC16A7*. One of the pleiotropic SNPs on chromosome 14, rs11157241, is located approx. 30 kbp upstream of *LRFN5*.

GTeX eQTL lookups revealed that some of these phenotype-associated SNPs were also associated with expression levels of nearby genes. A SNP associated only with MDD, on chromosome 1 (rs35641559), was found to be significantly associated with expression of a long non-coding RNA gene *LINC01360* in the testis (FDR < 0.05, Supplementary Table 6c). The MDD-associated and pleiotropic SNPs on chromosome 14 are all significantly associated with expression of *LRFN5* in a range of tissues, including brain, heart, adipose tissue and spleen (Supplementary Table 6c). The CPG-associated SNPs on chromosome 12 were not significantly associated with expression of any gene in the eQTL database (Supplementary Table 6c).

**Fig. 1:**
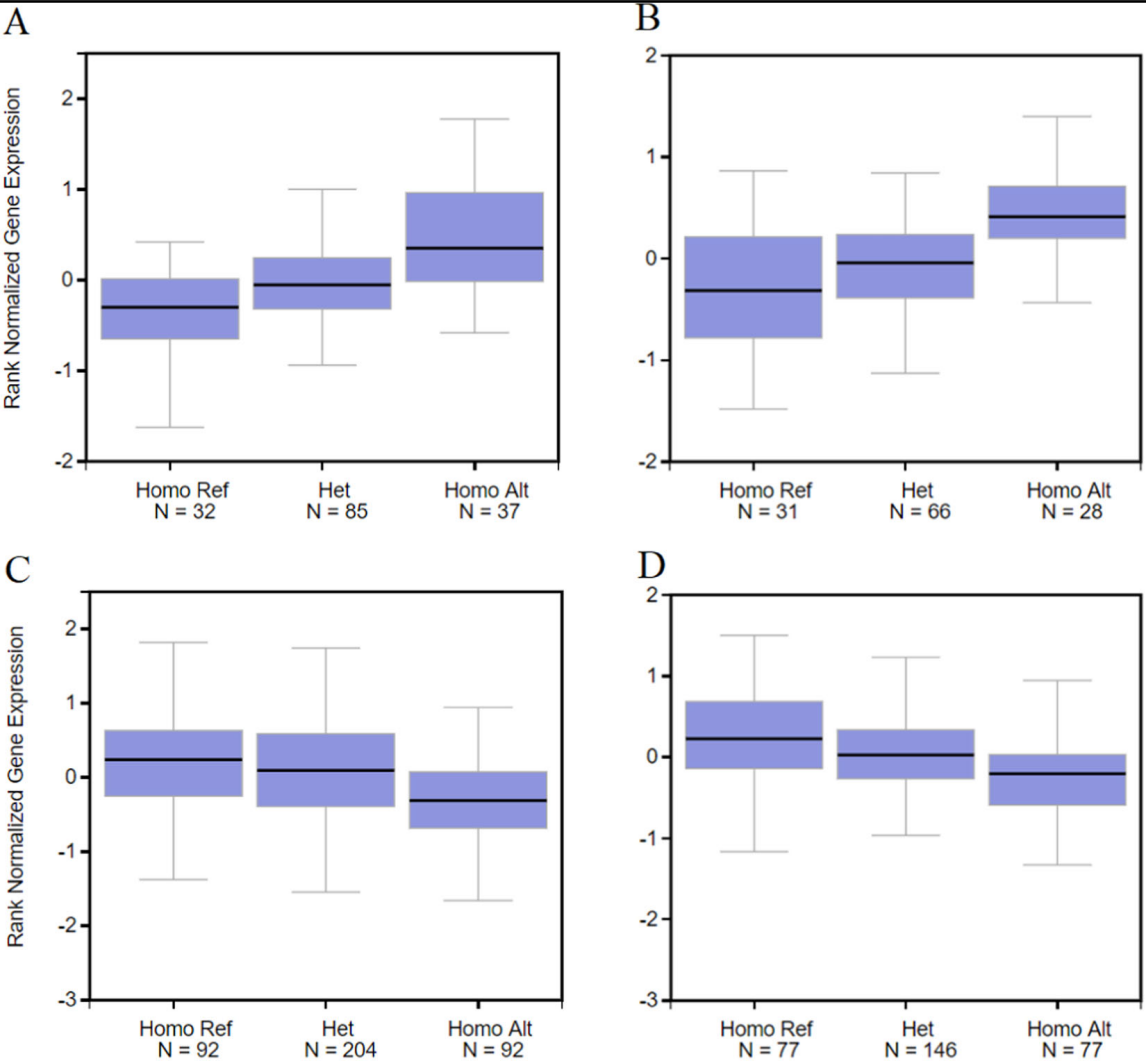
Expression of LRFN5 in brain and periphery. Boxplot output from single-tissue eQTL lookups (GTEx IGV eQTL Browser) of rs11846556, showing rank normalised gene expression values for *LRFN5*. **a** Cerebellum, **b** cerebellar hemisphere, **c** tibial artery and **d** transformed fibroblasts.

Single-tissue eQTL lookups of rs11846556 showed different trends in expression pattern of *LRFN5* with 0, 1 and 2A alleles, with a trend towards increased expression in the cerebellum and cerebellar hemisphere associated with homozygosity for the A allele (Fig. 1a, b respectively), and decreased expression in tibial artery and transformed fibroblasts associated with increasing number of copies of the A allele at this SNP locus (Fig. 1c, d respectively).

### Association between rs11846556 genotype and MDD and chronic pain in UK Biobank

To further examine pleiotropic variants within *LRFN5*, regression models were used to assess the strength of association between rs11846556 genotype and each of the two phenotypes of interest, chronic pain and MDD. Genotype at this SNP was associated with significantly increased risk for chronic pain (Table 2A; FDR-adjusted *p* = 0.0008), with each additional copy of the A allele increasing the odds of having chronic pain by 2% (OR = 1.02). rs11846556 genotype was also associated with chronic pain category (Table 2B), with each additional copy of the A allele associated with an OR of 1.02 per chronic pain category (FDR-adjusted *p* = 4.4 × 10^−5^). Genotype at rs11846556 was not significantly associated with MDD (Table 2A) or with MDD severity (Table 2B). rs11846556 genotype accounted for 0.29% of the variance in chronic pain presence and 0.25% of the variance in chronic pain category (estimated using McFadden’s *R*^2^, adjusting for age and sex). rs11846556 genotype was no longer associated with presence of chronic pain after adjustment for MDD status (FDR-adj *p* = 0.096), but the adjustment did yield a larger effect size (0.017 vs 0.015 in the MDD-adjusted and non-adjusted models, respectively).

**Table 2:** Analysis of associations between rs11846556 genotype and chronic pain/MDD.

	Beta	SE	OR	*p*	FDR-adj. *p*	Additional adjuster
(A)						
CP (dichot.)	0.015	0.004	1.02	2.63E-04	7.89E-04	
CP (dichot.)	0.017	0.008	1.02	0.048	0.096	MDD (dichot)
MDD	0.014	0.009	1.01	ns		
MDD	0.011	0.010	1.01	ns		CP (dichot)

	Beta	SE	*p*	FDR-adj. *p*		
(B)						
CPC	0.018	0.004	7.26E-06	4.36E-05		
MDD severity	0.014	0.009	ns			

(A) results of logistic regression models with dichotomous chronic pain (CP(dichot.)) and major depressive disorder (MDD) variables as outcomes and SNP genotype as predictor. SE = standard error of the beta coefficient value, OR = odds ratio. Ns = non-significant (*p* > 0.05)

(B) results of ordinal regression models with chronic pain category and MDD severity variables as outcomes and SNP genotype as predictor. SE = standard error of the beta coefficient, ns = non-significant (*p* > 0.05)

## Discussion

### CPG-associated SNPs

The SNPs associated with CPG only were located close to *SLC16A7*, which encodes monocarboxylate transporter 2 (MCT2). In the central nervous system, MCT2 is involved in high affinity, proton-coupled transport of metabolites (particularly lactate) into neurons and may play a role in neuronal uptake of energy substrates released by glia^57,58^. MCT2 is localised to the post-synaptic compartment in many human neurons and may have a specialised role in synaptic functioning^59,60^. Regulation of *SLC16A7* has also been linked to disorders of the brain: loss or under-expression has been associated with temporal-lobe epilepsy^61^ and it may be expressed and methylated at different levels in psychotic patients versus controls^62^.

### MDD-associated SNPs

The SNP on chromosome 1 associated with MDD only was located close to *LRRIQ3* and *FPGT*. *LRRIQ3* encodes leucine-rich repeat (LRR) and IQ motif containing protein 3, a calcium-channel component. LRR-domain containing proteins in general are involved in cell-cell communication, including processes involved in innate immunity and neuronal development^63,64^. *FPGT* encodes fucose-1-phosphate guanylyltransferase, a protein involved in the alternative (salvage) pathways of fucose metabolism^65^. Fucose metabolism is important in a variety of cell-cell communication and host-microbe interaction situations, but is also important during neuronal development^65^. Variants in the *LRRIQ3* region have been previously associated with schizophrenia^66^, neurodevelopmental disorders^67^ and migraine^68^.

### Pleiotropic SNPs in *LRFN5*

The significantly associated SNPs on chromosome 14 found within or close to *LRFN5* were either associated with MDD only or were pleiotropic and associated with both CPG and MDD. Due to the LD-based pruning carried out before the cFDR analysis, the MDD-only and pleiotropic SNPs are not in strong linkage disequilibrium and may thus be tagging different functional variants, potentially acting on risk in different ways. *LRFN5* encodes leucine-rich repeat (LRR) and fibronectin type 3 domain-containing protein 5. Proteins in the LRFN family span the plasma membrane, and their extracellular domains are thought to participate in the cell-cell interactions necessary for both neuronal development^69,70^ and synapse formation^71^. Lrfn5, along with another member of the Lrfn protein family, Lrfn2, may induce both inhibitory and excitatory presynaptic differentiation in nearby neuronal cells^72^, a process that may play a critical role in brain development and function^73^. As a family, these genes are primarily expressed in the central nervous system. Polymorphic markers within or close to *LRFN5* have been reported to be associated with progressive autism and familial schizophrenia^74,75^. Reduced expression of Lrfn5 has also been reported to contribute to neuro-inflammation^76^. Each of the four pleiotropic SNPs has opposing directions of effect in MDD and CPG. For example, the effect allele at rs11846556 is associated with an increase in CPG but with reduced risk of MDD. This may be explained by structural and connectivity-related changes in the brain which facilitate development and by the possibility that the maintenance of chronic pain might involve neurogenesis and synaptic plasticity^35,41,77^, whereas impaired neurogenesis is associated with depression^78,79^. Additionally, differing expression patterns of *LRFN5* in the brain and CNS versus the periphery may be involved in the opposing direction of effect associated with pleiotropic SNPs. Finally, as the SNPs were non-significantly associated in the original studies (conventional GWAS analyses of CPG) this means that although effect sizes differ in direction the confidence intervals for effect estimates of these SNPs on CPG will include zero, and may therefore not be truly opposing directions of effect. cFDR analyses using p values indicate pleiotropy in terms of significant cFDR-derived association, rather than informing on or calculating new effect sizes.

### Cis-eQTLs

SNPs within and close to *LRFN5* on chromosome 14, and in the region close to long non-coding RNA gene *LINC01360* on chromosome 1, were found to be cis-eQTLs. rs35641559 on chromosome 1, associated with MDD only, was found to be significantly associated with *LOC105378800* expression levels in the testis. All four pleiotropic SNPs on chromosome 14, were found to be significantly associated with *LRFN5* expression levels in a range of tissues, including the brain. Further investigation of potential regulatory roles for these or other nearby variants is warranted.

### Role of rs11846556 in the biology of chronic pain and depression

Pleiotropy broadly refers to genetic variants (such as SNPs) being associated with effects in more than one trait^52^. This pleiotropy can be either biological (also known as horizontal) or mediated (also known as vertical). Biological pleiotropy refers to genetic variants being associated with effects in two or more traits via independent mechanisms or pathways, whereas mediated pleiotropy describes a genetic variant associated with an effect in one trait, which then is associated with changes in a second trait—in other words the changes in the second trait are only associated with the genetic variant through the variant’s association with the first trait.

Number of A alleles at rs11846556, one of the pleiotropic SNPs identified here, was found to be associated with risk of chronic pain (and with higher values of the chronic pain category phenotype), but not with MDD risk or severity (in the UK Biobank cohort), and the effect size for the chronic pain association was not attenuated by inclusion of MDD in the regression model. This relationship is consistent with mediated pleiotropy rather than biological pleiotropy, i.e., a pathway in which genotype influences chronic pain risk, which in turn affects MDD risk, rather than influencing both by separate mechanisms. Formal mediation analyses was beyond the scope of this study primarily due to probable sequential ignorability violation and unmeasured confounders^80,81^.

As discussed above, each of the 4 SNPs tagged as pleiotropic, including rs11846556, are also cis-eQTLs for *LRFN5*. The SNP we explored in more detail, rs11846556, is a cis-eQTL of *LRFN5* in the cerebellum and cerebral hemisphere, along with tibial artery (GTEx single-tissue eQTL lookup). The number of A alleles at this SNP is significantly associated with increased expression of *LRFN5* in CNS tissues, but with a trend towards decreased expression in peripheral tissues (Fig. 1a–d), suggesting the existence of tissue-specific regulatory effects. This SNP is also a cis-eQTL for expression of a long intergenic non-coding RNA (Gencode ID: ENSG00000258636.1) on the reverse strand with respect to *LRFN5*, but only in spleen and aorta.

Alterations in synaptic plasticity and neurogenesis in the hippocampus, which is often non-normal in cases of neuropathic pain, may underlie some of the cognitive and affective deficits seen in patients with major depression^82^. However, in cases where depression is a downstream consequence of chronic pain, its onset may be influenced by lifestyle and other ‘above-the-skin’ factors that also operate downstream of chronic pain, rather than as a more direct result of synaptic changes related to *LRFN5* expression levels.

Our data therefore suggest that rs11846556 genotype is significantly associated with chronic pain, but not with MDD, and that the relationship between chronic pain and genotype is not attenuated by MDD—pleiotropic effects of related to rs11846556 on chronic pain and MDD appear to be mediated rather than biological. Possession of more copies of the A allele at this locus is associated with increased *LRFN5* expression levels in the CNS, which may exert its effect on transition to a chronic pain state after a prior insult through consequent alterations to brain network connectivity.

### Strengths and limitations

A key strength of this study lies in its enhanced power, using the cFDR approach, to detect the contribution of genetic variants to chronic pain relative to previous genome-wide approaches. Limitations include the self-reported nature of the chronic pain phenotype. In addition, the questions asked in the two studies were slightly different, generating chronic pain measures that may be capturing different aspects of the overall phenotype. ‘CPG’ as assessed in the Pfizer-23andMe GWAS may be capturing a more direct severity-related phenotype, whereas ‘chronic pain category’ as assessed in UKB is likely to be capturing a different aspect of chronic pain susceptibility related to sensitivity of the response to multiple insults, or to propensity to report. Despite this subtle distinction, we were able to demonstrate that an *LRFN5* SNP is associated with both these phenotypic pain measures.

## Conclusions

In this study six novel SNPs were found to be associated with CPG, a measure of pain severity. cFDR analyses also increased power to find MDD-associated variants in comparison to genome-wide significance thresholds. ccFDR analyses revealed evidence of pleiotropic effects of variants in *LRFN5*. The regions around the significant SNPs contain genes involved in, amongst other processes, neuronal cell metabolism and development, innate immunity and cell-cell interaction. These regions have also been previously implicated in a range of neurodevelopmental and psychiatric disorders. One of the pleiotropic SNPs identified on chromosome 14 (rs11846556), which is a cis-eQTL of *LRFN5* in cerebellum and cerebral hemisphere, was found to be associated with chronic pain but not MDD in UK Biobank. This provides intriguing preliminary evidence that the pleiotropy associated with variation in *LRFN5* may be mediated rather than horizontal. Additionally, although the genetic correlation between depression and chronic pain in terms of CPG^7^ and comorbidity have been previously reported, this study highlights a specific locus, possible mechanisms, and further detail on pleiotropy between the two conditions.

## Supplementary information

Supplementary Table S1

Supplementary Table S2

Supplementary Table S3

Supplementary Table S4

Supplementary Table S5

Supplementary Table S6a

Supplementary Table S6b

Supplementary Table S6c

## Data Availability

R script used to calculate cFDR and ccFDR values is derived from Liley & Wallace with modifications (as overlapping sample size was not a concern for this analysis)^48^, and is available from the authors upon request.
