# Supplementary material for: Identification of novel common variants associated with chronic pain using conditional false discovery rate analysis with major depressive disorder and assessment of pleiotropic effects of LRFN5
Source: Transl Psychiatry. 2019 Nov 20;9:310. doi: 10.1038/s41398-019-0613-4 (PMC6868167; doi:10.1038/s41398-019-0613-4)
Supplement: Supplementary file 2 — Supplementary Table S2 [file 41398_2019_613_MOESM2_ESM.docx]

| **Chronic Pain Category** | **Total** | **Male** | **Female** | **Mean Age** |
| --- | --- | --- | --- | --- |
| 0 | 265224 | 127870 | 137354 | 56.3 |
| 1 | 107603 | 49360 | 58243 | 56.7 |
| 2 | 75887 | 30673 | 45214 | 57.0 |
| 3 | 13889 | 4764 | 9125 | 56.8 |
| 4 | 6650 | 2415 | 4235 | 57.1 |

Demographic information on UK Biobank participants included in regression analyses of chronic pain category (total N = 469, 253).
