# Supplementary material for: Identification of novel common variants associated with chronic pain using conditional false discovery rate analysis with major depressive disorder and assessment of pleiotropic effects of LRFN5
Source: Transl Psychiatry. 2019 Nov 20;9:310. doi: 10.1038/s41398-019-0613-4 (PMC6868167; doi:10.1038/s41398-019-0613-4)
Supplement: Supplementary file 3 — Supplementary Table S3 [file 41398_2019_613_MOESM3_ESM.docx]

| **MDD Severity** | **Total** | **Male** | **Female** | **Mean Age** |
| --- | --- | --- | --- | --- |
| 0 | 89779 | 44256 | 45523 | 56.72 |
| 1 | 12813 | 4239 | 8574 | 54.88 |
| 2 | 19694 | 5711 | 13983 | 53.76 |

Demographic information on UK Biobank participants in regression analyses of MDD Severity (total N = 122, 286).
