# Supplementary material for: Identification of novel common variants associated with chronic pain using conditional false discovery rate analysis with major depressive disorder and assessment of pleiotropic effects of LRFN5
Source: Transl Psychiatry. 2019 Nov 20;9:310. doi: 10.1038/s41398-019-0613-4 (PMC6868167; doi:10.1038/s41398-019-0613-4)
Supplement: Supplementary file 4 — Supplementary Table S4 [file 41398_2019_613_MOESM4_ESM.docx]

| **Chronic Pain Category** | **Total** | **% MDD 1** | **% MDD 2** | **Male** | **Female** | **Mean Age** |
| --- | --- | --- | --- | --- | --- | --- |
| 0 | 75725 | 9.4 | 12.0 | 35361 | 40364 | 56.0 |
| 1 | 26795 | 11.5 | 17.7 | 11643 | 15152 | 56.1 |
| 2 | 15714 | 13.2 | 26.8 | 5955 | 9759 | 56.2 |
| 3 | 2257 | 13.9 | 43.7 | 639 | 1618 | 55.9 |
| 4 | 755 | 13.6 | 46.2 | 237 | 518 | 56.4 |

Demographic information on UK Biobank participants in regression analyses of chronic pain and MDD (total N = 121, 246). % MDD 1 = percentage with MDD severity = 1, % MDD 2 = percentage with MDD severity = 2.
