# Supplementary material for: Identification of novel common variants associated with chronic pain using conditional false discovery rate analysis with major depressive disorder and assessment of pleiotropic effects of LRFN5
Source: Transl Psychiatry. 2019 Nov 20;9:310. doi: 10.1038/s41398-019-0613-4 (PMC6868167; doi:10.1038/s41398-019-0613-4)
Supplement: Supplementary file 5 — Supplementary Table S5 [file 41398_2019_613_MOESM5_ESM.docx]

| snp | chr | cfdr_mdd1 | cfdr_cpg1 | conj |
| --- | --- | --- | --- | --- |
| rs8015100 | 14 | 9.27E-07 | 0.001 | 0.001 |
| rs11157241 | 14 | 1.28E-06 | 0.001 | 0.001 |
| rs10131184 | 14 | 7.10E-06 | 0.001 | 0.001 |
| rs11846556 | 14 | 3.76E-05 | 0.001 | 0.001 |
| rs1584317 | 14 | 0.0035 | 0.029 | 0.029 |
| rs4904790 | 14 | 0.004 | 0.020 | 0.020 |
| rs10138559 | 14 | 0.005 | 0.108 | 0.108 |
| rs10872954 | 14 | 0.007 | 0.053 | 0.053 |
| rs35641559 | 1 | 0.009 | 0.106 | 0.106 |
| rs71419096 | 2 | 0.011 | 0.037 | 0.037 |
| rs4143896 | 14 | 0.011 | 0.037 | 0.037 |
| rs4543157 | 4 | 0.018 | 0.505 | 0.505 |
| rs149981001 | 12 | 0.018 | 0.001 | 0.018 |
| rs10890031 | 1 | 0.019 | 0.164 | 0.164 |
| rs147573737 | 12 | 0.023 | 0.002 | 0.023 |
| rs7531118 | 1 | 0.028 | 0.517 | 0.517 |
| rs2958188 | 18 | 0.029 | 0.506 | 0.506 |
| rs12718437 | 1 | 0.032 | 0.250 | 0.250 |
| rs11627826 | 14 | 0.033 | 0.122 | 0.122 |
| rs10938179 | 4 | 0.035 | 0.740 | 0.740 |
| rs10078807 | 5 | 0.035 | 0.780 | 0.780 |
| rs11210247 | 1 | 0.036 | 0.264 | 0.264 |
| rs77415097 | 2 | 0.036 | 0.125 | 0.125 |
| rs10052804 | 5 | 0.040 | 0.788 | 0.788 |
| rs2581430 | 4 | 0.042 | 0.877 | 0.877 |
| rs78676209 | 5 | 0.044 | 0.969 | 0.969 |
| rs1592754 | 5 | 0.044 | 0.842 | 0.842 |
| rs833421 | 9 | 0.044 | 0.108 | 0.108 |
| rs396755 | 5 | 0.044 | 0.899 | 0.899 |
| rs116578037 | 5 | 0.047 | 0.918 | 0.918 |
| rs325506 | 5 | 0.050 | 1.013 | 1.013 |
| rs7528238 | 1 | 0.052 | 0.182 | 0.182 |
| rs1923243 | 1 | 0.053 | 0.289 | 0.289 |
| rs1582419 | 5 | 0.056 | 1.094 | 1.094 |
| rs4650196 | 1 | 0.060 | 0.227 | 0.227 |
| rs114773469 | 2 | 0.061 | 0.212 | 0.212 |
| rs7524355 | 1 | 0.061 | 0.198 | 0.198 |
| rs36057735 | 6 | 0.061 | 0.390 | 0.390 |
| rs148547443 | 10 | 0.063 | 0.775 | 0.775 |
| rs4593761 | 1 | 0.064 | 0.207 | 0.207 |
| rs11210177 | 1 | 0.065 | 0.352 | 0.352 |
| rs12127301 | 1 | 0.066 | 0.201 | 0.201 |
| rs12118352 | 1 | 0.068 | 0.204 | 0.204 |
| rs4491023 | 1 | 0.070 | 0.199 | 0.199 |
| rs56006809 | 7 | 0.078 | 0.225 | 0.225 |
| rs2286643 | 5 | 0.078 | 0.158 | 0.158 |
| rs7099700 | 10 | 0.079 | 0.841 | 0.841 |
| rs11845823 | 14 | 0.079 | 0.284 | 0.284 |
| rs16992132 | 4 | 0.082 | 0.276 | 0.276 |
| rs4129350 | 1 | 0.084 | 0.360 | 0.360 |

**cFDR Lookup Table.** snp = rsID, chr = chromosome, cfdr_mdd1 = cFDR value for MDD as primary trait, cfdr_cpg1 = cFDR value for CPG as primary trait, conj = ccFDR value.
