# Supplementary material for: Identification of novel common variants associated with chronic pain using conditional false discovery rate analysis with major depressive disorder and assessment of pleiotropic effects of LRFN5
Source: Transl Psychiatry. 2019 Nov 20;9:310. doi: 10.1038/s41398-019-0613-4 (PMC6868167; doi:10.1038/s41398-019-0613-4)
Supplement: Supplementary file 7 — Supplementary Table S6b [file 41398_2019_613_MOESM7_ESM.docx]

| rsID | Chromosome | cFDR-Associated Trait | Gene(s) |
| --- | --- | --- | --- |
| rs35641559 | 1 | MDD | *LINC01360, LRRIQ3, FPGT, FPGT-TNNI3K* |
| rs149981001 | 12 | CPG | *SLC16A7* |
| rs147573737 | 12 | CPG | *SLC16A7* |
| rs4904790 | 14 | MDD | *LRFN5* |
| rs1584317 | 14 | MDD | *LRFN5* |
| rs11846556 | 14 | Both | *LRFN5* |
| rs10131184 | 14 | Both | *LRFN5* |
| rs8015100 | 14 | Both | *LRFN5* |
| rs11157241 | 14 | Both | *LRFN5* |
| rs10138559 | 14 | MDD | *LRFN5* |
| rs10872954 | 14 | MDD | *LRFN5* |

**UCSC Genome Browser Search Results**.
